# (2-Chloro-8-methyl­quinolin-3-yl)methanol

**DOI:** 10.1107/S1600536810020490

**Published:** 2010-06-05

**Authors:** S. Mohana Roopan, F. Nawaz Khan, Rajesh Kumar, Venkatesha R. Hathwar, Mehmet Akkurt

**Affiliations:** a Organic and Medicinal Chemistry Research Laboratory, Organic Chemistry Division, School of Advanced Sciences, VIT University, Vellore 632 014, Tamil Nadu, India; b Solid State and Structural Chemistry Unit, Indian Institute of Science, Bangalore 560 012, Karnataka, India; c Department of Physics, Faculty of Arts and Sciences, Erciyes University, 38039 Kayseri, Turkey

## Abstract

The mol­ecule of title compound, C_11_H_10_ClNO, is close to being planar (r.m.s deviation for the non-H atoms = 0.017 Å). In the crystal, mol­ecules inter­act by way of O—H⋯O hydrogen bonds, generating *C*(2) chains propagating in [010]. The crystal structure is consolidated by C—H⋯π inter­actions and aromatic π–π stacking inter­actions [centroid–centroid distance = 3.661 (2) Å].

## Related literature

For a related structure and background references, see: Roopan *et al.* (2010 ▶). For a similar structure, see: Khan *et al.* (2009 ▶).
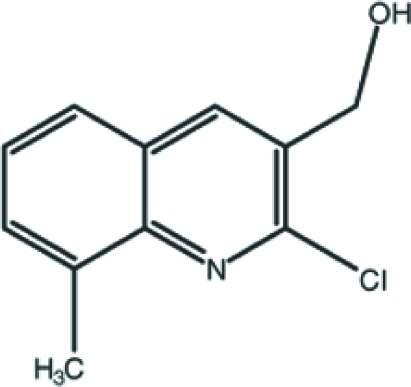


## Experimental

### 

#### Crystal data

C_11_H_10_ClNO
*M* *_r_* = 207.65
Monoclinic, 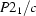

*a* = 14.963 (2) Å
*b* = 4.632 (1) Å
*c* = 14.469 (2) Å
β = 103.612 (1)°
*V* = 974.7 (3) Å^3^
*Z* = 4
Mo *K*α radiation
μ = 0.35 mm^−1^
*T* = 290 K
0.40 × 0.24 × 0.11 mm

#### Data collection

Oxford Xcalibur Eos(Nova) CCD detector diffractometer
Absorption correction: multi-scan (*CrysAlis PRO* RED; Oxford Diffraction, 2009 ▶) *T*_min_ = 0.871, *T*_max_ = 0.962
7607 measured reflections
1723 independent reflections
790 reflections with *I* > 2σ(*I*)
*R*_int_ = 0.167

#### Refinement

*R*[*F* ^2^ > 2σ(*F* ^2^)] = 0.061
*wR*(*F* ^2^) = 0.135
*S* = 0.85
1723 reflections
129 parameters
H-atom parameters constrained
Δρ_max_ = 0.23 e Å^−3^
Δρ_min_ = −0.23 e Å^−3^

### 

Data collection: *CrysAlis PRO CCD* (Oxford Diffraction, 2009 ▶); cell refinement: *CrysAlis PRO CCD*; data reduction: *CrysAlis PRO RED* (Oxford Diffraction, 2009 ▶); program(s) used to solve structure: *SHELXS97* (Sheldrick, 2008 ▶); program(s) used to refine structure: *SHELXL97* (Sheldrick, 2008 ▶); molecular graphics: *ORTEP-3* (Farrugia, 1997 ▶); software used to prepare material for publication: *WinGX* (Farrugia, 1999 ▶).

## Supplementary Material

Crystal structure: contains datablocks global, I. DOI: 10.1107/S1600536810020490/hb5470sup1.cif

Structure factors: contains datablocks I. DOI: 10.1107/S1600536810020490/hb5470Isup2.hkl

Additional supplementary materials:  crystallographic information; 3D view; checkCIF report

## Figures and Tables

**Table 1:** Hydrogen-bond geometry (Å, °) *Cg*1 is a centroid of the N1/C1–C3/C8/C9 ring.

*D*—H⋯*A*	*D*—H	H⋯*A*	*D*⋯*A*	*D*—H⋯*A*
O1—H1*O*⋯O1^i^	0.82	1.90	2.712 (4)	174
C10—H10*A*⋯*Cg*1^ii^	0.97	2.75	3.557 (4)	141

Symmetry codes: (i) 
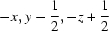
; (ii) 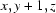.

**Fig. 1.**
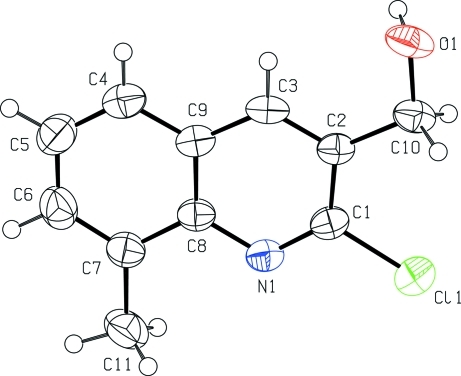
The molecule of (I), showing 50% probability displacement ellipsoids.

**Fig. 2.**
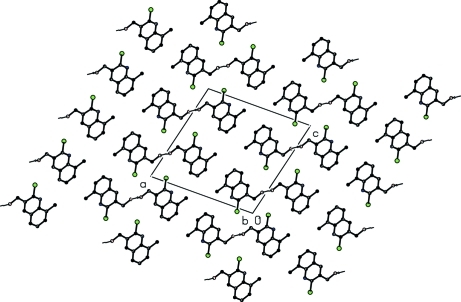
A view of the packing of (I) with intermolecular O–H···O hydrogen bonding down the b axis. The H atoms not involved in hydrogen bonds have been omitted for clarity.

## supplementary crystallographic information

### Comment

As part of our program which aimed to develop new selective and environmentally friendly methodologies for the preparation of 2-chloroquinolines (Roopan *et al.*,  2010), we report here crystal structure of the title compound, (I).

The title molecule (I), (Fig. 1), except the hydroxyl and methyl H atoms, close to planar (r.m.s deviation 0.017 Å). The values of the geometric parameters in (I) are comparable to those of some similar structures (Khan *et al.*, 2009).

In the solid-state, the molecules are linked *via* intermolecular O—H···O hydrogen bonds (Table 1, Fig. 2). The crystal structure is further stabilized by an intermolecular C–H···π interactions between the methylene H atom of ethenol substituent and the pyridine ring of an adjacent molecule, with a C10–H10A···*Cg*1^ii^ separation of 2.75 Å (Table 1, *Cg*1 is the centroid of N1/C1–C3/C8/C9 pyridine ring; symmetry code: (ii) *x*, *y* + 1, *z*). In addition, the packing mode results in stabilizing π-π stacking interactions [*Cg*1···*Cg*2^ii^ = 3.661 (2) Å, where *Cg*1 and *Cg*2 are the centroids of the N1/C1–C3/C8/C9 and C4–C9 rings].

### Experimental

2-Chloro-8-methylquinoline-3-carbaldehyde (206 mg, 1 mmol), sodium borohydride (38 mg, 1 mmol) and catalytic amount of montmorillonite K-10 were taken in an open vessel and the resulting mixture was irradiating at 500 W for 4 min. Ethylacetate was poured into the reaction mixture and filtered off. The filtrated after removal of solvent ethy lacetate was subjected to column chromatography packed with silica and ethyl acetate/petroleum ether was used as the eluant.  Colourless plates of (I) were grown by solvent evaporation from a solution of the compound in chloroform.

### Refinement

H atoms were positioned geometrically, with C—H = 0.93- 0.97 Å, and refined a riding model with *U*_iso_(H) = 1.2 or 1.5 *U*_eq_(C). The value of *R*_int _[0.167] is greater than 0.12, which may reflect the poor crystal quality.

### Crystal data

**Table:**

C_11_H_10_ClNO	*F*(000) = 432
*M**_r_* = 207.65	*D*_x_ = 1.415 Mg m^−^^3^
Monoclinic, *P*2_1_/*c*	Mo *K*α radiation, λ = 0.71073 Å
Hall symbol: -P 2ybc	Cell parameters from 972 reflections
*a* = 14.963 (2) Å	θ = 2.0–20.5°
*b* = 4.632 (1) Å	µ = 0.35 mm^−^^1^
*c* = 14.469 (2) Å	*T* = 290 K
β = 103.612 (1)°	Plate, colourless
*V* = 974.7 (3) Å^3^	0.40 × 0.24 × 0.11 mm
*Z* = 4	

### Data collection

**Table:**

Oxford Xcalibur Eos(Nova) CCD detector diffractometer	1723 independent reflections
Radiation source: Enhance (Mo) X-ray Source	790 reflections with *I* > 2σ(*I*)
graphite	*R*_int_ = 0.167
ω scans	θ_max_ = 25.0°, θ_min_ = 2.9°
Absorption correction: multi-scan (*CrysAlis PRO* RED; Oxford Diffraction, 2009)	*h* = −17→17
*T*_min_ = 0.871, *T*_max_ = 0.962	*k* = −5→5
7607 measured reflections	*l* = −17→17

### Refinement

**Table:**

Refinement on *F*^2^	Primary atom site location: structure-invariant direct methods
Least-squares matrix: full	Secondary atom site location: difference Fourier map
*R*[*F*^2^ > 2σ(*F*^2^)] = 0.061	Hydrogen site location: inferred from neighbouring sites
*wR*(*F*^2^) = 0.135	H-atom parameters constrained
*S* = 0.85	*w* = 1/[σ^2^(*F*_o_^2^) + (0.0492*P*)^2^] where *P* = (*F*_o_^2^ + 2*F*_c_^2^)/3
1723 reflections	(Δ/σ)_max_ < 0.001
129 parameters	Δρ_max_ = 0.23 e Å^−^^3^
0 restraints	Δρ_min_ = −0.23 e Å^−^^3^

### Special details

**Table:**

Geometry. Bond distances, angles *etc*. have been calculated using the rounded fractional coordinates. All su's are estimated from the variances of the (full) variance-covariance matrix. The cell e.s.d.'s are taken into account in the estimation of distances, angles and torsion angles
Refinement. Refinement on *F*^2^ for ALL reflections except those flagged by the user for potential systematic errors. Weighted *R*-factors *wR* and all goodnesses of fit *S* are based on *F*^2^, conventional *R*-factors *R* are based on *F*, with *F* set to zero for negative *F*^2^. The observed criterion of *F*^2^ > σ(*F*^2^) is used only for calculating -*R*-factor-obs *etc*. and is not relevant to the choice of reflections for refinement. *R*-factors based on *F*^2^ are statistically about twice as large as those based on *F*, and *R*-factors based on ALL data will be even larger.

### Fractional atomic coordinates and isotropic or equivalent isotropic displacement parameters (Å^2^)

**Table:**

	*x*	*y*	*z*	*U*_iso_*/*U*_eq_	
Cl1	0.13301 (8)	0.6900 (2)	−0.03546 (7)	0.0599 (5)	
O1	0.0342 (2)	0.8800 (5)	0.2237 (2)	0.0534 (11)	
N1	0.2487 (2)	0.3588 (7)	0.0784 (2)	0.0372 (11)	
C1	0.1808 (3)	0.5392 (8)	0.0749 (2)	0.0368 (14)	
C2	0.1436 (3)	0.6216 (7)	0.1526 (3)	0.0340 (14)	
C3	0.1835 (3)	0.4990 (8)	0.2376 (3)	0.0398 (16)	
C4	0.2981 (3)	0.1674 (9)	0.3335 (3)	0.0475 (17)	
C5	0.3681 (3)	−0.0242 (10)	0.3370 (3)	0.0551 (17)	
C6	0.3985 (3)	−0.0855 (9)	0.2554 (3)	0.0538 (17)	
C7	0.3611 (3)	0.0351 (9)	0.1686 (3)	0.0427 (17)	
C8	0.2875 (3)	0.2323 (8)	0.1643 (3)	0.0365 (12)	
C9	0.2566 (3)	0.3003 (8)	0.2465 (3)	0.0369 (14)	
C10	0.0635 (3)	0.8293 (8)	0.1397 (3)	0.0436 (16)	
C11	0.3954 (3)	−0.0328 (10)	0.0817 (3)	0.0589 (17)	
H1O	0.00970	0.73400	0.23830	0.0800*	
H3	0.16230	0.54700	0.29100	0.0480*	
H4	0.27790	0.20970	0.38810	0.0570*	
H5	0.39560	−0.11390	0.39410	0.0660*	
H6	0.44670	−0.21540	0.26010	0.0640*	
H10A	0.08110	1.01170	0.11630	0.0520*	
H10B	0.01230	0.75250	0.09200	0.0520*	
H11A	0.44600	−0.16520	0.09810	0.0880*	
H11B	0.41540	0.14190	0.05710	0.0880*	
H11C	0.34680	−0.11840	0.03440	0.0880*	

### Atomic displacement parameters (Å^2^)

**Table:**

	*U*^11^	*U*^22^	*U*^33^	*U*^12^	*U*^13^	*U*^23^
Cl1	0.0614 (8)	0.0749 (9)	0.0457 (7)	0.0113 (7)	0.0170 (6)	0.0132 (6)
O1	0.060 (2)	0.0369 (17)	0.078 (2)	0.0016 (16)	0.0457 (18)	−0.0011 (16)
N1	0.042 (2)	0.035 (2)	0.0375 (19)	−0.0031 (18)	0.0153 (17)	−0.0004 (17)
C1	0.044 (3)	0.031 (2)	0.037 (2)	−0.007 (2)	0.013 (2)	−0.0008 (19)
C2	0.040 (3)	0.028 (2)	0.037 (2)	−0.005 (2)	0.015 (2)	−0.003 (2)
C3	0.044 (3)	0.043 (3)	0.039 (2)	−0.009 (2)	0.023 (2)	−0.006 (2)
C4	0.051 (3)	0.052 (3)	0.042 (3)	−0.010 (3)	0.016 (2)	0.000 (2)
C5	0.053 (3)	0.063 (3)	0.047 (3)	−0.004 (3)	0.007 (2)	0.012 (2)
C6	0.038 (3)	0.051 (3)	0.071 (3)	−0.001 (2)	0.010 (3)	0.011 (3)
C7	0.037 (3)	0.044 (3)	0.048 (3)	−0.006 (2)	0.012 (2)	0.002 (2)
C8	0.037 (2)	0.036 (2)	0.038 (2)	−0.007 (2)	0.012 (2)	−0.002 (2)
C9	0.043 (3)	0.036 (2)	0.034 (2)	−0.007 (2)	0.014 (2)	−0.003 (2)
C10	0.046 (3)	0.038 (2)	0.053 (3)	−0.004 (2)	0.024 (2)	−0.002 (2)
C11	0.050 (3)	0.069 (3)	0.063 (3)	0.010 (3)	0.024 (2)	−0.006 (3)

### Geometric parameters (Å, °)

**Table:**

Cl1—C1	1.735 (3)	C7—C11	1.499 (6)
O1—C10	1.406 (5)	C7—C8	1.421 (6)
O1—H1O	0.8200	C8—C9	1.409 (6)
N1—C8	1.373 (5)	C3—H3	0.9300
N1—C1	1.307 (5)	C4—H4	0.9300
C1—C2	1.419 (6)	C5—H5	0.9300
C2—C10	1.514 (6)	C6—H6	0.9300
C2—C3	1.359 (6)	C10—H10A	0.9700
C3—C9	1.412 (6)	C10—H10B	0.9700
C4—C9	1.408 (6)	C11—H11A	0.9600
C4—C5	1.365 (6)	C11—H11B	0.9600
C5—C6	1.391 (6)	C11—H11C	0.9600
C6—C7	1.368 (6)		
			
C10—O1—H1O	110.00	O1—C10—C2	113.5 (3)
C1—N1—C8	117.8 (3)	C2—C3—H3	119.00
Cl1—C1—N1	116.2 (3)	C9—C3—H3	119.00
Cl1—C1—C2	117.9 (3)	C5—C4—H4	120.00
N1—C1—C2	126.0 (3)	C9—C4—H4	120.00
C1—C2—C3	115.7 (4)	C4—C5—H5	120.00
C1—C2—C10	121.3 (4)	C6—C5—H5	120.00
C3—C2—C10	122.9 (4)	C5—C6—H6	118.00
C2—C3—C9	121.4 (4)	C7—C6—H6	118.00
C5—C4—C9	119.4 (4)	O1—C10—H10A	109.00
C4—C5—C6	120.2 (4)	O1—C10—H10B	109.00
C5—C6—C7	123.4 (4)	C2—C10—H10A	109.00
C6—C7—C11	122.5 (4)	C2—C10—H10B	109.00
C8—C7—C11	120.9 (4)	H10A—C10—H10B	108.00
C6—C7—C8	116.6 (4)	C7—C11—H11A	109.00
N1—C8—C9	121.0 (4)	C7—C11—H11B	109.00
C7—C8—C9	120.8 (4)	C7—C11—H11C	109.00
N1—C8—C7	118.2 (4)	H11A—C11—H11B	109.00
C3—C9—C4	122.4 (4)	H11A—C11—H11C	109.00
C4—C9—C8	119.6 (4)	H11B—C11—H11C	110.00
C3—C9—C8	118.0 (4)		
			
C8—N1—C1—Cl1	−179.2 (3)	C9—C4—C5—C6	0.5 (7)
C8—N1—C1—C2	1.0 (6)	C5—C4—C9—C3	179.6 (4)
C1—N1—C8—C7	179.8 (4)	C5—C4—C9—C8	0.4 (6)
C1—N1—C8—C9	−1.5 (6)	C4—C5—C6—C7	−0.7 (7)
Cl1—C1—C2—C3	−179.7 (3)	C5—C6—C7—C8	0.0 (7)
Cl1—C1—C2—C10	1.3 (5)	C5—C6—C7—C11	179.6 (4)
N1—C1—C2—C3	0.1 (6)	C6—C7—C8—N1	179.6 (4)
N1—C1—C2—C10	−178.9 (4)	C6—C7—C8—C9	0.9 (6)
C1—C2—C3—C9	−0.7 (6)	C11—C7—C8—N1	0.0 (6)
C10—C2—C3—C9	178.3 (4)	C11—C7—C8—C9	−178.7 (4)
C1—C2—C10—O1	178.4 (3)	N1—C8—C9—C3	1.0 (6)
C3—C2—C10—O1	−0.6 (5)	N1—C8—C9—C4	−179.8 (4)
C2—C3—C9—C4	−179.1 (4)	C7—C8—C9—C3	179.7 (4)
C2—C3—C9—C8	0.1 (6)	C7—C8—C9—C4	−1.1 (6)

### Hydrogen-bond geometry (Å, °)

**Table:**

Cg1 is a centroid of the N1/C1–C3/C8/C9 ring.

**Table:**

*D*—H···*A*	*D*—H	H···*A*	*D*···*A*	*D*—H···*A*
O1—H1O···O1^i^	0.82	1.90	2.712 (4)	174
C10—H10A···Cg1^ii^	0.97	2.75	3.557 (4)	141

Symmetry codes: (i) −*x*, *y*−1/2, −*z*+1/2; (ii) *x*, *y*+1, *z*.
